# Decay Dynamics of Tumors

**DOI:** 10.1371/journal.pone.0157689

**Published:** 2016-06-16

**Authors:** Álvaro G. López, Jesús M. Seoane, Miguel A. F. Sanjuán

**Affiliations:** Nonlinear Dynamics, Chaos and Complex Systems Group. Departamento de Física, Universidad Rey Juan Carlos, Tulipán s/n, 28933 Móstoles, Madrid, Spain; IUMPA - Universitat Politecnica de Valencia, SPAIN

## Abstract

The *fractional cell kill* is a mathematical expression describing the rate at which a certain population of cells is reduced to a fraction of itself. We investigate the mathematical function that governs the rate at which a solid tumor is lysed by a cell population of cytotoxic lymphocytes. We do it in the context of enzyme kinetics, using geometrical and analytical arguments. We derive the equations governing the decay of a tumor in the limit in which it is plainly surrounded by immune cells. A cellular automaton is used to test such decay, confirming its validity. Finally, we introduce a modification in the fractional cell kill so that the expected dynamics is attained in the mentioned limit. We also discuss the potential of this new function for non-solid and solid tumors which are infiltrated with lymphocytes.

## Introduction

The oversimplification of cancer as the growth of an independent subset of rebel mutated cells within a tissue presents great difficulties explaining tumor development [1, 2]. The relative importance of the dynamics at the tissue level, represented by the interactions of the tumor cells with their environment, compared to the role played by mutations, is still a subject of intense debate [3, 4]. The tumor microenvironment includes stromal cells (*e.g*. immune cells, fibroblasts or endothelial cells), the extracellular matrix, and signalling molecules such as cytokines or growth factors. The particular cellular and molecular mechanisms, as well as their role in tumor development, are complex and not sufficiently well understood [5]. Even though all of them might prove to be important in the fight against cancer, immunotherapy is lately focusing great attention. Probably, this is because the immune system is better known and has evolved for centuries to neatly destroy threatening foreign organisms in our body. Therefore, there is evidence and hope that it can be trained to effectively destroy tumor cells, which originate in the body, as well. However, given the complexity of biological systems and the ubiquity of nonlinearity at all scales, it is hardly believable that this task can be rigorously achieved without the guidance of mathematical models. Positively, these models, together with the experimental ones, might provide the guiding principles that biologists precise to unveil the counter-intuitive nature of the complex interactions among cancer cells and their environment during the evolution of tumors. This, in turn, might permit clinicians to design better treatments for the vast variety of cancers [6].

In the context of tumor-immune interactions, it is worth and interesting to analyse the potential that enzyme kinetics offers [7, 8]. Enzymatic reactions can be viewed in an abstract manner as an asymmetric interaction between two entities, one being rather passive (the substrate) and the other being rather active (the enzyme). When these two entities make contact, the latter affects the former transforming it into some other entity (the product). Thus, an enzymatic reaction can be casted in three steps: the formation of a complex from the two parts, a subsequent transformation of the passive part by its active counterpart and their final dissociation. As long as these conditions are fullfilled, there is no general reason preventing us to use this conceptual framework not only at the chemical scale, but also at the cellular scale and, perhaps, even at higher scales. For example, the growth of microorganisms in the presence of a limited substrate obeys the Michaelis-Menten kinetics [9]. In ecology, the intake rate of a consumer as a function of the density of preys is also a kinetics of this type [10]. In all these cases, whenever there is a considerable imbalance between the number of active and passive elements, saturation occurs. This is due to the limited capacity of the active part to interact with a sufficiently high number of elements of the passive counterpart. Note that this is also true in the reverse direction, since the passive elements can not interact with an enormous number of active elements for short times. In other words, interactions occur locally and require some time.

Inspired by this reasoning, a mathematical model describing tumor-immune interactions was designed by Kuznetsov *et al*. [11] to explore a possible dynamical origin of the dormancy and the sneaking-through of tumors. In their original model, the rate at which a tumor is lysed, *i.e*., the fractional cell kill, increases linearly with the number of immune cells, just as in an ordinary Lotka-Volterra model [12, 13]. Simply put, the velocity at which a tumor is destroyed can be increased unboundedly by simply adding more immune cells. Nevertheless, their work served as a foundation for other works concerning the interactions between immune and tumor cells [14, 15]. Among these works, a mathematical model was validated using experiments from mice [16] and men [17]. To reproduce the experimental data, these authors proposed a new fractional cell kill for the lysis of tumor cells by CD8^+^ lymphocytes. They noticed that the lysis curves seen in experimental settings exhibited saturation. Briefly, the fraction of lysed tumor cells after a certain time (usually a few hours in chromium release assays) versus different values of the initial effector-to-target ratio saturates for increasing values of the latter. Therefore, they proposed a Hill function [18, 19] depending on the effector-to-target ratio as the mathematical function describing the rate at which a tumor is lysed. Their brilliant achievement notwithstanding, little theoretical explanation was given to this function and the original proposal [11] was partly forgotten.

We have developed a simpler model [15], validated it and proposed several hypotheses to explain the nature of the fractional cell kill. Later on [20], the enzymatic conceptual framework was retaken showing that such hypotheses are sensible. We modified the mathematical function so that now the parameters have a clear biological significance and the function can be derived analytically. This function demonstrates that the speed at which a tumor is reduced by the immune system is governed by a modified Michaelis-Menten kinetics, where the rates of the reaction depend as power laws on the enzyme concentration. In the present work, we further explore this mathematical expression by examining the different limits that it provides. To reproduce also the time series as well as the lysis curves, we introduce one last rearrangement, which we believe makes it theoretically more conspicuous.

## Materials and Methods

### Tumor lysis kinetics

**Fig 1:**
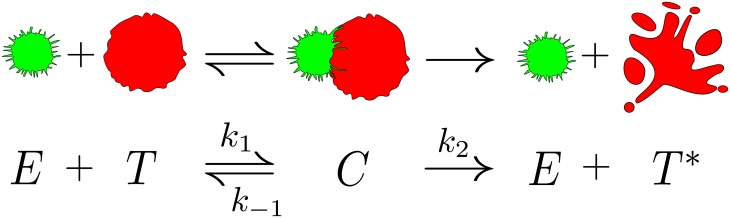
The cell-mediated immune response as an enzymatic reaction. An interaction between an activated lymphocyte *E*, colored in green, and a tumor cell *T*, painted in red. When the lymphocyte identifies the tumor cell these two cells form a complex. The result of the interaction is the initial T cell and an apoptotic tumor cell T*. This cellular interaction is similar to an enzymatic chemical reaction, where the tumor cell plays the role of the substrate and the T cell acts as an enzyme.

Using the Michaelis-Menten kinetics as the modelling framework describing tumor-immune interactions at the cellular scale [11], a mathematical expression describing the velocity at which a population of cytotoxic cells lyse a tumor has been derived in a previous work [20]. According to this model, the process of tumor cell lysis is equivalent to an enzymatic reaction where the tumor cells correspond to the substrate and the immune cells correspond to the enzyme. A schematic representation of such a cellular reaction is seen in Fig 1. When a T cell identifies a tumor cell through the recognition of antigens, these two cells form complexes. As a result, apoptosis is induced and a dead tumor cell is produced. However, some of the assumptions that lead to the Michaelis-Menten kinetics, such as a high substrate concentration compared to the enzyme concentration, or high values of the Michaelis constant compared to the enzyme concentration, are not met in the present case. To reproduce experiments, the constant rates of the reaction require dependence on the number of effector cells, in such a manner that saturation of the velocity is also found for increasing numbers of the effector cells. As previously stated, saturation occurs in both directions. The differential equation [20] describing the velocity at which the tumor cells are destroyed is $\dot{T}=-K\left(E,T\right)T$, with *K*(*E*, *T*) the fractional cell kill, which can be written as

$$\begin{array}{c} K\left(E,T\right)=d\frac{E^{\lambda }}{sT+E^{\lambda }}, \end{array}$$ (1)

where *T* and *E* represent the number of tumor cells and immune cells respectively. The parameters *d* and *λ* depend on the tumor geometry. Less spherical tumors lead to higher values of these parameters. On the other hand, the parameter *s* is related to the intrinsic ability of the cytotoxic cells to recognize and destroy their adversaries. Smaller values of this parameter are related to more effective immune cells. Thus, the velocity at which a tumor is lysed is given by

$$\begin{array}{c} \dot{T}=-d\frac{E^{\lambda }}{sT+E^{\lambda }}T. \end{array}$$ (2)

This equation states that the velocity at which a tumor is lysed by a population of cytotoxic cells becomes faster as this immune cell population increases. However, once the vicinity of the tumor is vastly occupied with several layers of immune cells, the remaining immune cells are not in contact with their tumor adversaries, and saturation is attained. Even in a situation in which the tumor cells are considerably bigger than the immune cell, and several immune cells are in contact with a single tumor cell, the velocity at which the tumor is lysed is not substantially enhanced. Accordingly, the saturation process depends on the size of the tumor. The number of immune cells for which the fractional cell kill *K*(*E*, *T*) is half of its maximum *d* increases monotonically with the tumor burden. Concerning the tumor cell population, also faster lytic velocity occurs for bigger tumors, but again saturation befalls. Now the reason is that for a big tumor cell population compared to the immune cell population, at some point the addition of tumor cells can not increase the velocity at which the tumor is lysed, since these added tumor cells are not in contact with immune cells, which are already busy lysing some of the initial tumor cells.

**Fig 2:**
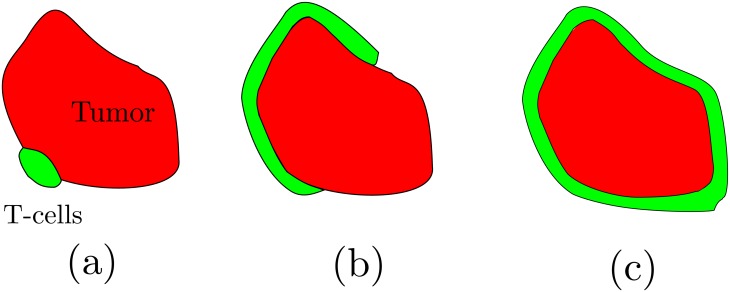
The limits of the fractional cell kill. (a) A small immune cell population facing a big tumor. In this limiting situation the decay of the tumor is rather linear, as shown in Eq (3). (b) The intermediate case in which a considerable part of the tumor is covered with immune cells. (c) A tumor which surface is totally covered with immune cells. In this extreme case the velocity of the decay can be approximated by a parabolic decay, as shown in Eq (6).

It is worth and interesting to carefully examine the different limits that this equation possesses (see Fig 2). For a fixed number of immune cells *E*_0_, when the immune cell population is small compared to the tumor size ($E_{0}^{\lambda }\ll sT$), the tumor cell population is reduced at a constant velocity

$$\begin{array}{c} \dot{T}=-dE_{0}^{\lambda }/s. \end{array}$$ (3)

**Fig 3:**
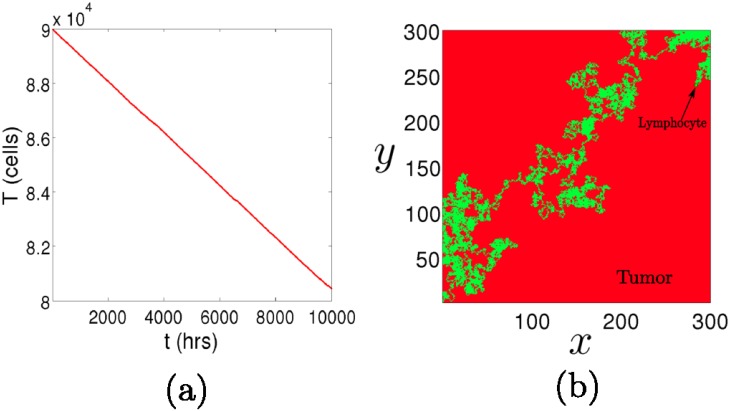
A single cell lysing a tumor. (a) The linear decay of a tumor in the limit in which there is only one immune cell. (b) The path (green) of a lymphocyte after a certain time, modelled by an unbiased random walk in a square domain, which is occupied by tumor cells (red). The initial condition is set on the left bottom corner.

This *linear decay* makes perfect sense if we bear in mind the extreme situation in which there is only one lymphocyte fighting a tumor of a certain size. Ideally, if it takes the immune cell approximately one hour to lyse a tumor cell, then the velocity of the decay is simply one tumor cell per hour. Even though this is fairly obvious, in Fig 3 we show the random walk of a lymphocyte lysing a tumor that occupies a square domain, at one cell per hour. In practice, the velocity clearly depends on the intrinsic ability of the cytotoxic cell *s* to lyse the tumor cells and also on the tumor morphology *λ* and *d*. On the other hand, when the immune cell population is high enough compared to the tumor cell population ($E_{0}^{\lambda }\gg sT$), Eq (2) yields an *exponential decay*

$$\begin{array}{c} \dot{T}=-dT. \end{array}$$ (4)

Now, the scenario corresponds to the case in which the tumor is totally covered with effector cells. For the sake of simplicity, we consider a tumor spheroid [21]. At each step the immune cells lyse a layer of tumor cells, and the radius of the spheroid decreases. In the next round another layer is eliminated but, since the tumor has smaller radius, so it does the length of this second layer. Therefore, the velocity decreases as the tumor is gradually erased. Nevertheless, note that for a three-dimensional solid tumor the reduction occurs in surface while the tumor is distributed in volume, suggesting that the decay should be slower than exponential.

**Fig 4:**
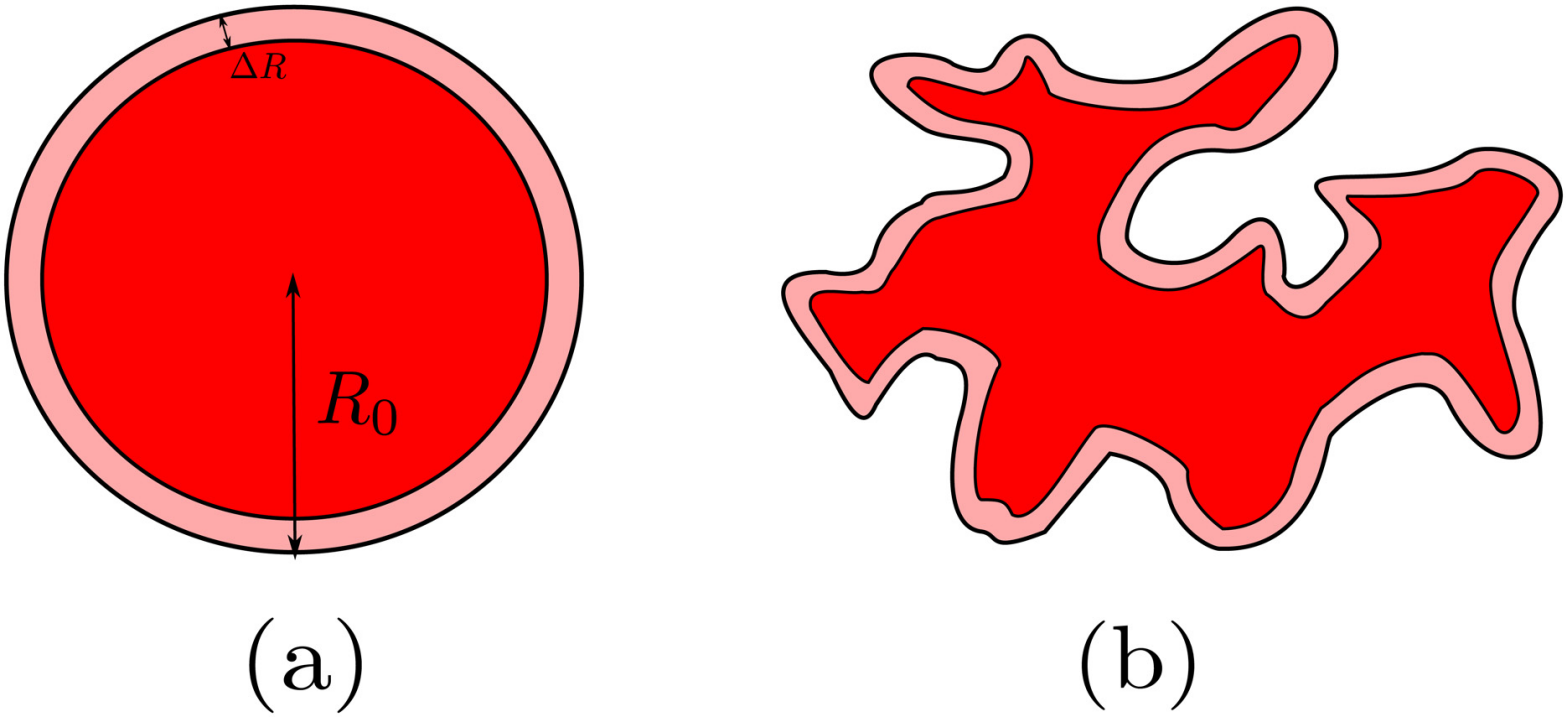
Two tumors with a destroyed layer. (a) A tumor with the shape of a disk and initial radius *R*_0_. At each step the immune system erases a layer (light red), reducing its radius by an amount Δ*R*. (b) Again a tumor with a destroyed layer, but exhibiting a more complex geometry.

It has been demonstrated [20] that Eq (2) reproduces accurately the values of the lysis after some fixed time versus different values of the effector-to-target ratio as initial conditions. However, here we show that it is unable to reproduce the time series of the tumor decay faithfully. A mathematical function which is good at reproducing the time series of the tumor decay can be derived in the following manner. Assume that a two-dimensional tumor with the shape of a disk is plainly covered with immune cells. As shown in Fig 4(a), a layer of tumor cells is erased by the immune cells at each step, like peeling an onion. If we write the radius of the disk at the *n*-th step as *R*_*n*_, and the diameter of a cell as Δ*R*, the dynamics of the tumor can be represented by a very simple map in the form *R*_*n*+1_ = *R*_*n*_ − Δ*R*. Since the area of a disk is related to the radius through *A* = *πR*^2^, a direct substitution yields the map $A_{n+1}=A_{n}+\pi \Delta R^{2}-2\pi^{1/2}\Delta RA_{n}^{1/2}$, where *A*_*n*_ is the area of the disk at the *n*-th step. If we consider that the immune cells lyse at a constant rate, then Δ*R* = *c*Δ*t*, and we obtain

$$\begin{array}{c} \frac{\Delta A_{n}}{\Delta t}=\pi c^{2}\Delta t-2\pi^{1/2}cA_{n}^{1/2}. \end{array}$$ (5)

Finally, assuming that the cell density of the tumor is approximately constant, that the tumor is big enough so that the time intervals can be considered infinitesimal and defining a decay constant as *d* = 2*π*^1/2^ *c*, we obtain the differential equation

$$\begin{array}{c} \dot{T}=-dT^{1/2}. \end{array}$$ (6)

More simply, if we consider a disk of area *A* = *πR*^2^ and assume that the velocity at which the radius decreases is constant $\dot{R}=-c$, with *c* > 0, we can write

$$\begin{array}{c} \frac{dA}{dt}=2\pi R\frac{dR}{dt}=-2\pi^{1/2}cA^{1/2}. \end{array}$$ (7)

If the tumor has a more sophisticated geometry, we can still apply Eq (6) under appropriate assumptions. Suppose that a layer of a tumor with complex geometry is erased by the immune system. At every step along the tumor decay, we can associate to the tumor mass a disk of equal area. Thus, the decay of the tumor is again equivalent to a sequence of disks with decreasing radii. Now, however, we can not guarantee that the radius decreases a fixed value at each step. To demonstrate this statement, it suffices to consider a pair of counterexamples. A very simple one arises if we consider a tumor with the shape of an ellipse. Assuming that the semi-major axis *a* and the semi-minor axis *b* both decrease at a constant rate $\dot{a}=\dot{b}=-c$, we find

$$\begin{array}{c} \frac{dA}{dt}=\pi \frac{da}{dt}b+\pi a\frac{db}{dt}=-\pi^{1/2}c\left[{\left(1-e^{2}\right)}^{1/4}+{\left(1-e^{2}\right)}^{-1/4}\right]A^{1/2}, \end{array}$$ (8)

where *e* = *e*(*t*) is the eccentricity of the ellipse, which changes over time. Note that for *e* = 0 we recover Eq (7).

Things get even more complicated if we take an initial tumor which is not a convex set, as the one depicted in Fig 4(b). Even in the case in which all the immune cells act synchronously and are equally effective, the topology of the tumor might change during the process of lysis, becoming disconnected. Assuming equal decay rates *d* and using Eq (6), it is straightforward to verify that the total area of two tumors with the shape of a disk does not decay as a whole with the same velocity than that of a single tumor with such shape and equal total area. The two small tumors decay faster, because the ratio between the perimeter and the enclosed area is larger. Analytically, this is simply a consequence of the nonlinear nature of Eq (6). Therefore, we designate the mean value of the variations of the radius of such sequence of disks as Δ*R*. Then, we write the variation of the radius as *δ*_*n*_Δ*R*, where *δ*_*n*_ accounts for the deviations with respect to the mean value, that must be bounded. The map is now *R*_*n*+1_ = *R*_*n*_ − *δ*_*n*_Δ*R* and the area goes as $A_{n+1}=A_{n}+\pi \delta_{n}^{2}\Delta R^{2}-2\pi^{1/2}\delta_{n}\Delta RA_{n}^{1/2}$. Making the same assumptions as in the previous case, the final result is

$$\begin{array}{c} \dot{T}=-d\left(t\right)T^{1/2}, \end{array}$$ (9)

where *d*(*t*) = 2*π*^1/2^*cδ*(*t*), and *δ*(*t*) a function which takes into account the deviations from Eq (6) due to the change in morphology and connectedness at each step. In the Results we show that these deviations due to a complex morphology are small for the connected tumors here examined. Therefore, the *parabolic decay* represented in Eq (6) works well at reproducing the decay of the tumors in the limit in which they are completely surrounded by immune cells, as long as they are not formed by disconnected pieces and their shape does not differ too much from a spherical shape. In the S1 Appendix we derive an explicit relation between *δ*(*t*) and the geometrical properties of the tumor. Also a more general differential equation representing the decay of a two-dimensional surface with a non-trivial shape and topology is developed.

### A cellular automaton model

**Fig 5:**
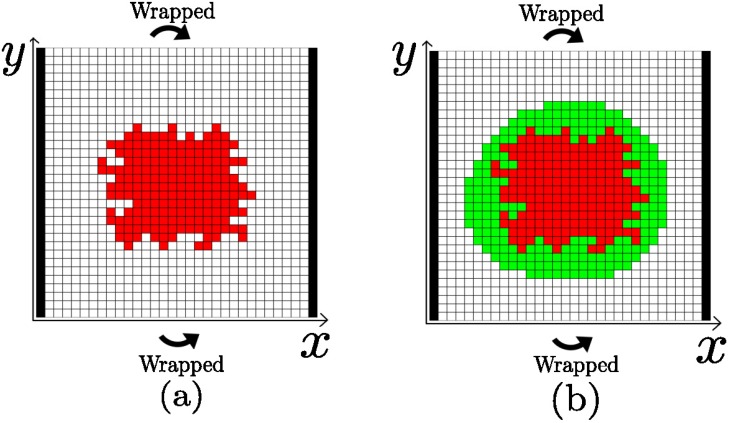
The cellular automaton. (a) A grid representing the cellular automaton during the growth of a tumor. The tumor cells are shown in red, while the remaining spots are occupied by healthy cells. The vertical black stripes in the boundary of the square domain represent the vessels from which nutrients diffuse, and periodic boundary conditions are considered in the remaining part of the boundary. (b) Once a tumor is grown up to a certain size, immune cells are placed around the tumor (green) and the lysis of the tumor is registered until it is totally eradicated. During this second step, the dynamics of the tumor is freezed, since we are only interested in the lysis.

A cellular automaton (CA) model [22, 23] for the growth of avascular tumors is used to study the velocity at which the tumors decay. An schematic representation of the CA is shown in Fig 5. The exact CA rules, as well as the equations for the diffusion of nutrients (such as glucose and oxygen) from the vessels are thoroughly described in a previous work [20]. We refer the reader to such work for a rigorous inspection. Here, we briefly describe its main features.

1. The cells are treated discretely, allowing them to occupy diverse grid points in a quadrilateral spatial domain. Three different types of cells are considered: healthy cells, tumor cells and immune cells.
2. The actions of these cells evolve according to probabilistic rules, that depend on some parameters and the nutrient concentration at each point of the grid. There is a probabilistic rule for each type of action *a*, that depends on a specific parameter *θ*_*a*_. This parameter represents the intrinsic capacity of the cells to carry out that particular action. The role of healthy cells is rather passive, consuming nutrients and being replaced by tumor and immune cells. Tumor cells can divide *θ*_*div*_, migrate *θ*_*mig*_ or die *θ*_*nec*_, while the immune cells can migrate, inactivate *θ*_*inc*_ or lyse *θ*_*lys*_. When this last action occurs, recruitment *θ*_*rec*_ is induced and new immune cells appear in their surrounding neighbourhood. Finally, the cells are not allowed to lyse more than three times, and become inactivated, disappearing from the region of interest.
3. The diffusion of nutrients from the vessels into such spatial region is represented through linear reaction-diffusion equations, which are continuous and deterministic. Two types of nutrients are utilized, distinguishing between those which are specific for cell division *N* and others that are related to other cellular activities *M*. The rate of consumption of nutrients are represented by the parameters *λ*_*M*_, *λ*_*N*_ and *α*. Dirichlet boundary conditions are imposed on the vertical sides of the domain, where the vessels are placed. The horizontal upper and lower bounds of the domain obey periodic boundary conditions.

As in previous works, the simulations are carried out in two successive steps. The first is devoted to the growth of the tumors. Then the immune cells are placed in the immediate domain of the tumor and we let the system evolve. We are only interested in the dynamics of the lysis of the tumor. Thus, during this second step, we freeze the tumor growth, as if the tumor cells had been irradiated. It is important to recall that the decay laws here investigated, given by Eqs (4) and (6), are deterministic and valid in the limit in which the tumor is totally covered by immune cells. Therefore, we assume that, as the successive layers of the tumor are lysed, the immune cells advance quickly towards the tumor. In our cellular automaton this can be guaranteed when the immune cells are placed isotropically covering the whole tumor through the mechanism of recruitment. We insist that it is only in this limiting situation that we can make use of the cellular automaton for comparison with Eq (6). After placing the cells as shown in Fig 5(b), those immune cells that are far from the tumor become inactivated (disappear from the region). As a result, what we see are several outer layers of immune cells lysing the tumor. However, when a few immune cells are placed at a particular location in the boundary of the tumor, stochastic effects associated to T cell inactivation and recruitment impose an appreciable deviation from the expected linear decay. Nevertheless, the tendency to linearity is observed as the initial number of effector cells becomes smaller.

## Results

### The effect of morphology

**Fig 6:**
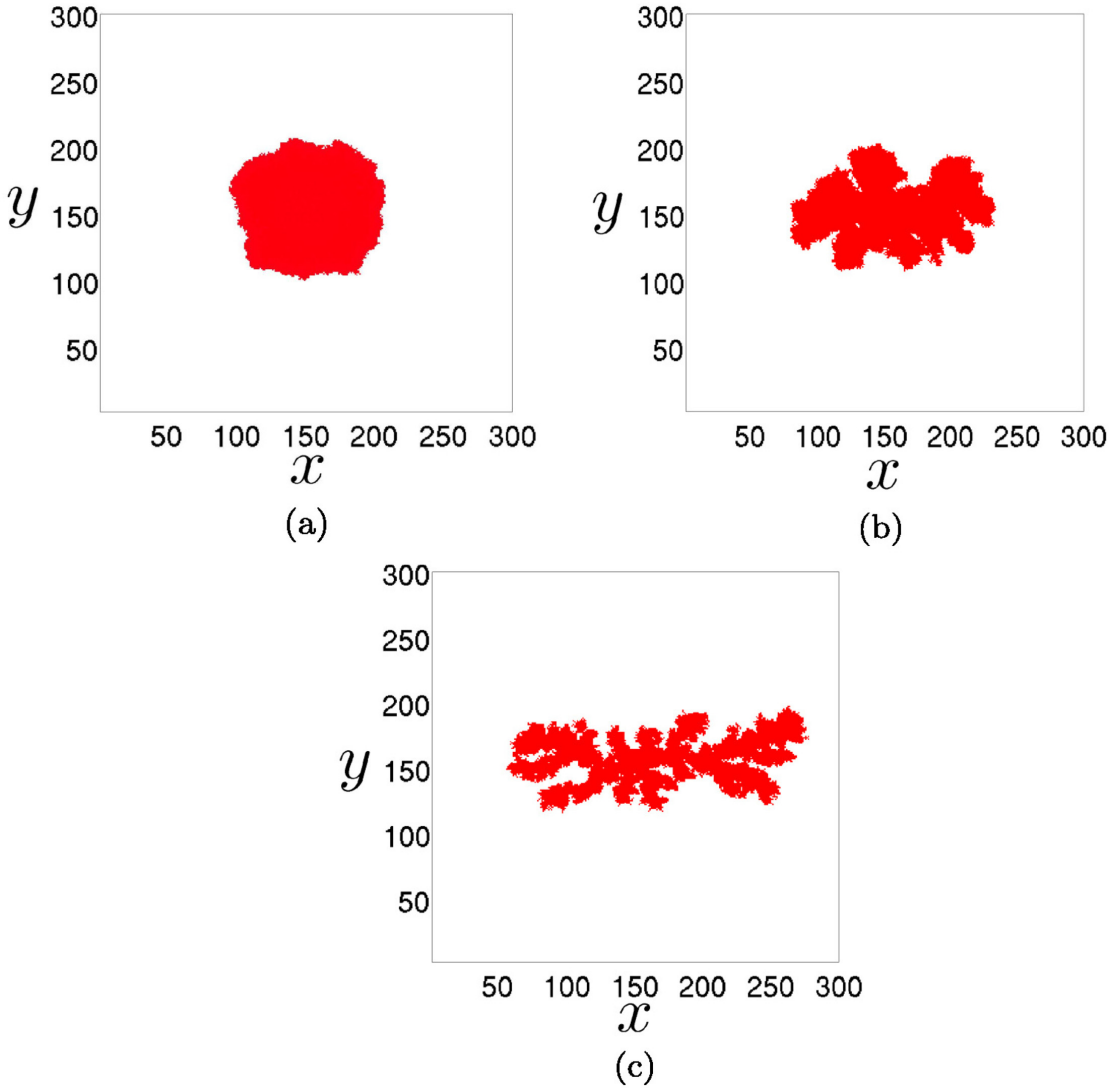
Three tumors grown by iteration of the cellular automaton. A grid of *n* × *n* cells, with *n* = 300 has been used. We disregard necrosis and motility of tumor cells by setting the parameters, *θ*_*nec*_ = 0 and *θ*_*mig*_ = ∞. In all the three cases *λ*_*M*_ = 10. (a) A spherical tumor obtained for parameter values *α* = 2/*n*, *λ*_*N*_ = 25 and *θ*_*div*_ = 0.3. (b) A papillary tumor obtained for parameter values *α* = 4/*n*, *λ*_*N*_ = 200 and *θ*_*div*_ = 0.3. (c) A filamentary tumor obtained for parameter values *α* = 8/*n*, *λ*_*N*_ = 270 and *θ*_*div*_ = 0.3. These three tumors have grown up to approximately 9100 cells.

**Fig 7:**
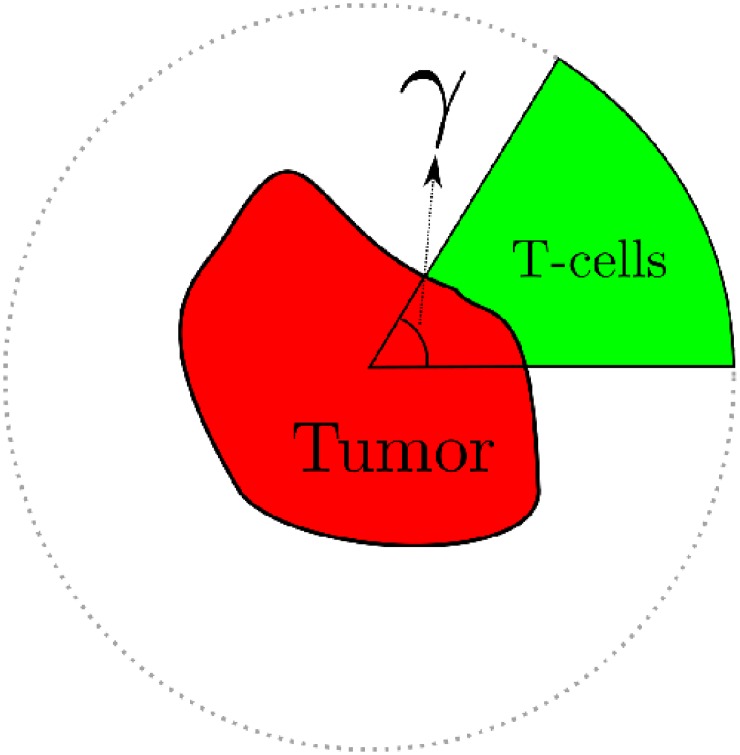
How the initial conditions are set to investigate the lysis of the different tumors. The tumors are inscribed in a circumference and immune cells are placed in the surroundings for different angles *γ*. Since those cells that are not close to the tumor outest layer become inactivated during the first steps of the CA, small values of the angle correspond to the case shown in Fig 2(a), while the case *γ* = 2*π* is related to Fig 2(c).

**Fig 8:**
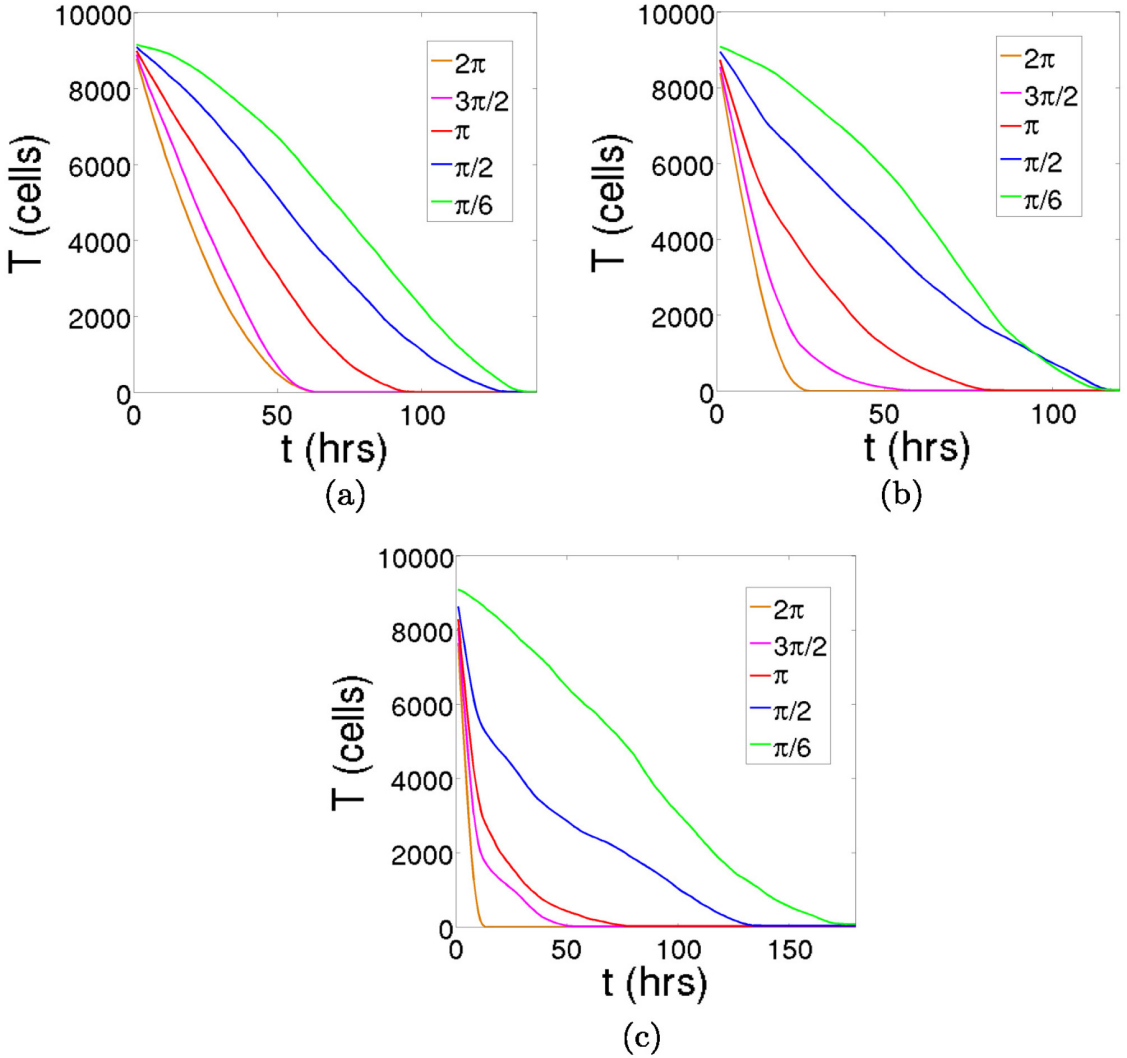
The decay of the three tumors for different initial conditions. The immune cells are placed in the neighborhood of the tumors for values of the angles *γ* = {*π*/6, *π*/2, *π*, 3*π*/2, 2*π*} and we iterate the CA. The CA actions corresponding to the lymphocytes have parameter values *θ*_*lys*_ = 0.3, *θ*_*rec*_ = 0.5 and *θ*_*inc*_ = 0.5. The tumor cells dynamics has been frozen, and the parameters related to the diffusion of nutrients are the same as those appearing in previous figures. (a) The decay of the spherical tumor for the different initial conditions. (b) The decay of the papillary tumor for the different initial conditions. (c) The decay of the filamentary tumor for the different initial conditions. As less immune cells are placed in the vicinity of the tumors as initial conditions (from *γ* = 2*π* to *γ* = *π*/6), the parabolic decay transforms into a more or less linear type of decay.

We use the cellular automaton model to inspect three different morphologies of two-dimensional tumors: a spherical tumor, a papillary tumor and filamentary tumor. The tumors generated with the cellular automaton are shown in Fig 6. We place these three tumors inside a circumference and, for each of them, we repeat the experiments for several initial conditions. To this end, we fill with immune cells the remaining space of the circumference for increasing angles, as depicted in Fig 7. The time series representing the decay of the tumors are shown in Fig 8. As explained in previous sections, we see a tendency towards linearity as the tumor is initially less covered with immune cells. Even the curvature is inverted for such small values of the initial angle, but this is surely a consequence of recruitment in the cellular automaton. Note also that the stochastic effects are more noticeable when the number of initial effector cells is low.

**Fig 9:**
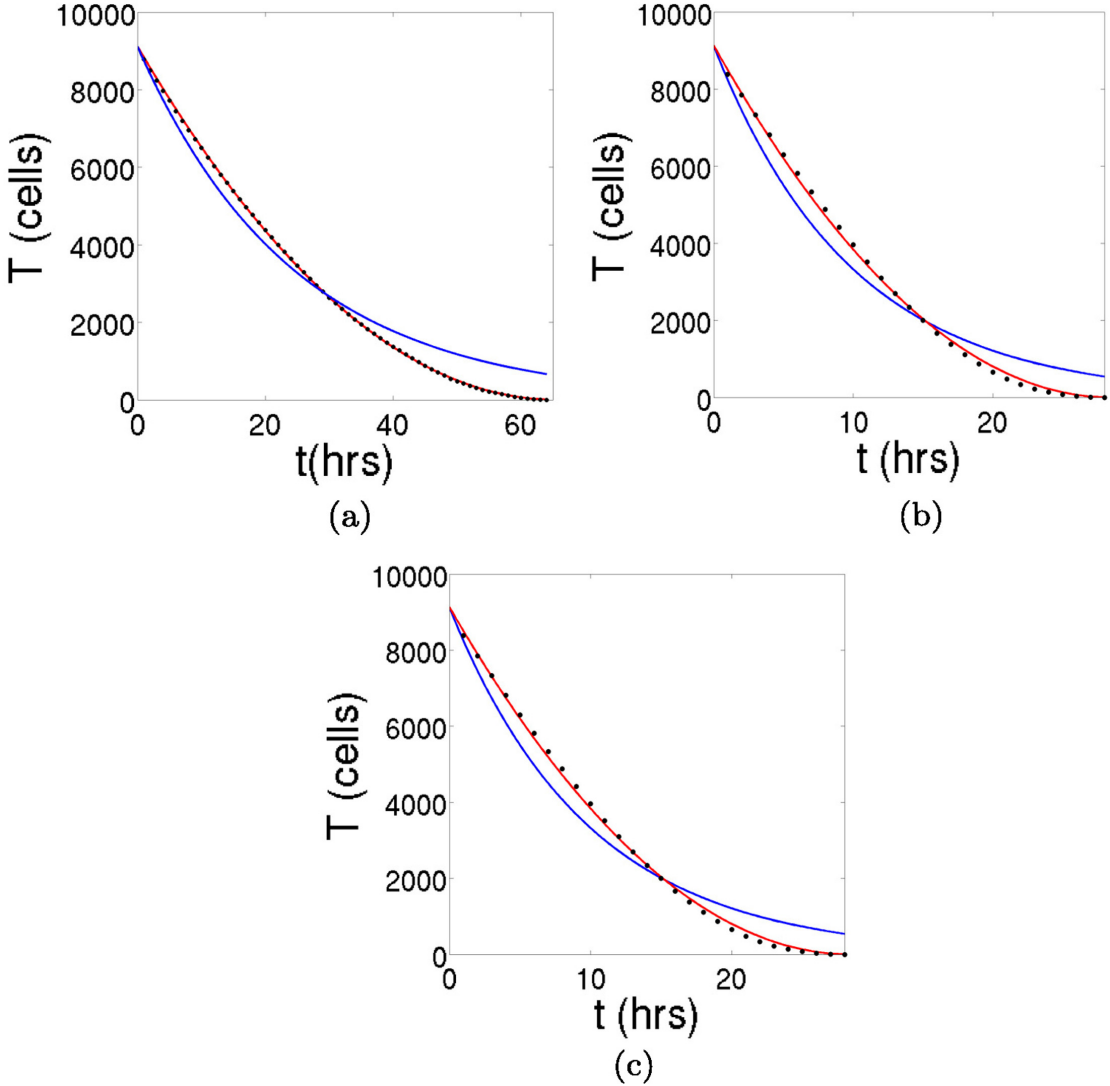
The decay of the three tumors for *γ* = 2*π*. We have iterated the cellular automaton in the limit in which the tumors are totally covered with immune cells. The results are fitted to a power-law function $\dot{T}=-dT^{\nu }$, shown in red, and an exponential decay $\dot{T}=-dT$, shown in blue, to elucidate which type of decay represents better the velocity with which the tumors shrink. (a) The decay of the spherical tumor. (b) The decay of the papillary tumor. (c) The decay of the filamentary tumor. In all the cases a power-law function with an approximate value of *ν* = 1/2 fits much better the results of the CA. Therefore, the decay is parabolic. The exact values are listed in Table 1.

The cases in which the tumors are totally covered with immune cells as initial conditions (*γ* = 2*π*) are fitted to the equation $\dot{T}=-dT^{\nu }$ and also to $\dot{T}=-dT$, to elucidate which type of decay represents better the tumor cell lysis. The parameters are obtained through a least square fitting method, and are listed in Table 1. As it can be seen in Fig 9, the exponential decay is much worse at describing the time evolution of this dynamical system. Moreover, the value of *ν* that gives the best fit to the power-law function is equal to one half for the papillary and the filamentary tumors, and practically one half for the spherical case. The agreement is striking and, as previously predicted, the fluctuations are higher when the tumors exhibit a more complex geometry. Concerning the parameter *d*, we see that more branchy tumors display higher values. The explanation for this behavior is evident, since the higher it is the contact surface of a tumor, the more cells that can interact with it and the faster the speed at which it is lysed. This is in conformity with results obtained in another work [20], where it was claimed that tumors with an spherical symmetry are harder to lyse. For a rigorous mathematical demonstration of this statement we refer the reader to the S1 Appendix. The crucial concept here is the accessibility that the immune cells have to the tumor cells.

**Table 1:** The parameter values of the decay.

Power-law decay			Exponential decay		
Parameter	Units	Value	Units	Value	Description
*d*(s)	cell^1/2^ hr^−1^	1.34	hr^−1^	0.04	Rate of decay
*d*(p)	cell^1/2^ hr^−1^ 3	3.36	hr^−1^	0.10	Rate of decay
*d*(f)	cell^1/2^ hr^−1^ 3	7.31	hr^−1^	0.21	Rate of decay
*ν*(s)		0.49			Exponent
*ν*(p)		0.50			Exponent
*ν*(f)		0.50			Exponent

The parameter values of the power-law decay $\dot{T}=-dT^{\nu }$ and the exponential decay $\dot{T}=-dT$, to which the data of the cellular automaton are fitted by means of a least-squares fitting method. We see that as the geometry of the tumor changes from spherical (s) through papillary (p) to filamentary (f), the parameter *d* increases. However, the value of *ν* is almost the same for the three geometries.

Thus, we have demonstrated that in the limit in which a solid tumor is totally covered with immune cells, the velocity at which it decays is slower than exponential. This fact requires modifying Eq (2) so that such limit is attained. The mathematical arguments previously employed can be perfectly extended to tumors that live in a three-dimensional space. If we recall that saturation of the velocity must be attained in the limit of infinitely big tumors, we propose that the kinetics of tumor lysis in the cell-mediated immune response to tumor growth is given by

$$\begin{array}{c} \dot{T}=-d\frac{E^{\lambda }}{sT^{\nu }+E^{\lambda }}T^{\nu }, \end{array}$$ (10)

where the exponent *ν* depends on the dimension of the space, the morphology of the tumor and its connectedness. For realistic, connected and rather spherical solid tumors we have *ν* = 2/3, with the 2 standing for surface, and the 3 for volume. However, in those cases in which the tumor is very disconnected and the immune cells are well mixed with the tumor cells, as for instance in haematological cancers or solid tumors profusely infiltrated with lymphocytes, *ν* = 1 should be used. The exponential decay arising in the limit $E_{0}^{\lambda }\gg sT$ would be then interpreted from a stochastic point of view, regarding the process as a Poisson process. Indeed, not all the immune cells have the same capacity to recognize a tumor cell, neither they act synchronously. In this case, the decay of a tumor does not differ substantially from other types of decay phenomena, as for example one-decay processes in radioactivity. For intermediate situations, the exponent *ν* will take a value between 2/3 and 1.

## Discussion

Our work demonstrates that the kinetics governing the lysis of a two-dimensional solid tumor that is infiltrated with lymphocytes ranges from linear to exponential. When there is no infiltration, the decay ranges from a linear decay to a parabolic decay. The linear decay corresponds to small values of the effector-to-target ratio as initial conditions, while the parabolic decay represents a tumor that is widely surrounded by immune cells. Intermediate situations are governed by Eq (10), which is a Hill function for both the tumor and the immune cell populations. The two exponents in this function are both smaller than or equal to one, representing the non-cooperative effects among tumor cells and immune cells that arise in the interaction as a consequence of geometry and cell crowding. To conclude this work, we also want to recall that when the immune cells are not effective recognizing the tumor cells (*s* → ∞), a linear decay results once again. As reasoned in other works [20], a large number of immune cells that “interact” ineffectively with a tumor can be considered equivalent to a small number of immune cells interacting effectively. To demonstrate this assertion mathematically, we recall that when a population of cytotoxic cells is ineffective lysing a tumor, only a fraction *f* of such cell population interacts with it. By inserting *fE* in Eq (10), we can redefine *s*/*f*^*λ*^ to be *s* and obtain the same equation.

## Supporting Information

S1 Appendix The decay of an arbitrary tumor. (PDF)
