# Supplementary material for: Decay Dynamics of Tumors
Source: PLoS One. 2016 Jun 16;11(6):e0157689. doi: 10.1371/journal.pone.0157689 (PMC4910984; doi:10.1371/journal.pone.0157689)
Supplement: S1 Appendix — (PDF) [file pone.0157689.s001.pdf]

Decay dynamics of tumors

Álvaro G. López[‡*], Jesús M. Seoane[‡], Miguel A. F. Sanjuán[‡],

Nonlinear Dynamics, Chaos and Complex Systems Group.
Departamento de Física, Universidad Rey Juan Carlos, Tulipán s/n, 28933 Móstoles, Madrid, Spain

‡These authors contributed equally to this work.
* alvaro.lopez@urjc.es

Supporting Information

S1 Appendix. The decay of an arbitrary tumor

We start with a convex set in $\mathbb{R}^2$, and assume that the boundary of such set is differentiable. We consider a solid tumor that is plainly covered with activated lymphocytes, in such a manner that the outmost layer of the tumor is erased at each step. Given a tumor with a particular boundary, we can approximate the T cells by small disks of diameter ΔR placed on such boundary. Then, perhaps, the most reasonable assumption is to consider that these cells reduce the surface in the normal direction to the boundary of the tumor, as depicted in Fig. 1.

At each step n of the process of lysis, the boundary can be represented by a parametric curve in the form $\gamma_n(\lambda) = (x_n(\lambda), y_n(\lambda))$, being λ the parameter used in such representation. Then, the decay can be mathematically expressed as a sequence of planar curves

$$\gamma_{n+1}(\lambda) = \gamma_n(\lambda) + \Delta R p_n(\lambda), \tag{1}$$

where $p_n(\lambda)$ is the normal unitary vector to $\gamma_n(\lambda)$. Recursive relations for the tangent and the normal vectors have to be found using the Frenet-Serret formulas, and a sequence of planar surfaces enclosed by these curves has to be defined. Finally, the element of area can be derived from the metric, its integral computed to obtain an equation for the variations of the area at each step, and a continuum limit worked out. These computations are very extensive, involve complicated integrals depending on the curvature at each step, and do not allow to draw clear conclusions.

Another possibility is to use polar coordinates, and consider that the initial boundary of the set can be represented through the curve $R_0(\theta)$. The area enclosed by this initial curve is computed as

$$A_0 = \frac{1}{2}\int_0^{2\pi} R_0^2(\theta) d\theta. \tag{2}$$

Now, we introduce the dependence on time, so that the initial area decreases in all directions at constant velocity. Mathematically, we have

$$A(t) = \frac{1}{2}\int_0^{2\pi} R^2(\theta, t) d\theta, \tag{3}$$

with the radius of the set decreasing with constant velocity in every direction, *i.e.*,

$$\frac{\partial R}{\partial t} = -c. \tag{4}$$

Note that now the surface is reduced in the radial direction, which is not normal to the boundary, except for a spherical tumor. Therefore, this approximation is worse than the one suggested in the first place. Nevertheless, it allows to derive continuous equations and draw neat conclusions in a very simpler manner. Generally, it takes a T cell around fourty minutes to lyse a tumor cell. If we approximate the diameter of a cell to 10μm, the value of c is around 10μm/hr. Integrating Equation (4) we can obtain the equation of motion representing the decay of the radius, which can be written in a simple manner as $R(\theta, t) = R_0(\theta) - ct$. This equation is only valid as long as the radius does not vary too much in different directions, compared to its size. Otherwise, the radius in a particular direction $R(\theta, t)$ could become zero (even negative) as other directions are still being erased. Thus, we assume that the condition $R_0(\theta) \gg |R_0'(\theta)|$ holds. Differentiating Equation (3) with respect to the time yields

$$\dot{A}(t) = -c \int_0^{2\pi} R(\theta, t) d\theta. \tag{5}$$

Substituting the equation of the radius in Equation (5) leads to

$$\dot{A}(t) = -c \int_0^{2\pi} R_0(\theta) d\theta + 2\pi c^2 t. \tag{6}$$

Under the condition previously imposed, the integral appearing in Equation (6) is approximately the length of the boundary of the initial set L_0. Thus, the differential equation governing the decrease of a convex set with a non-spherical shape can be approximated by

$$\dot{A}(t) = -cL_0 + 2\pi c^2 t. \tag{7}$$

It is straightforward to integrate this equation, if we consider the initial condition given by $A(0) = A_0$. The result is

$$A(t) = A_0 - cL_0 t + \pi c^2 t^2. \tag{8}$$

This equation represents a uniformly accelerated motion [1], where the initial velocity is $v_0 = -cL_0$, while the acceleration is $a = 2\pi c^2$. In other words, the decay is *parabolic*. Intuitively, if the radius decays linearly, the area does it quadratically. The time τ at which the tumor is totally erradicated can be computed by setting $A = 0$, which yields

$$\tau = \frac{L_0}{2\pi c} \left(1 - \sqrt{1 - 4\pi \frac{A_0}{L_0^2}} \right). \tag{9}$$

Note that the isoperimetric inequality [2] imposes $4\pi A_0 \leq L_0^2$ for any planar surface, with the inequality saturating for a disk. Thus, tumors with a spherical shape are the hardest to lyse. Since for a disk we have $L_0 = 2\pi^{1/2} A_0^{1/2}$, substitution in Equation (8) yields

$$A(t) = \left(A_0^{1/2} - \frac{1}{2}(2\pi^{1/2} c) t \right)^2. \tag{10}$$

This function is the solution to the differential equation $\dot{A} = -(2\pi^{1/2} c) A^{1/2}$, obtained in previous sections. However, for more complex morphologies this power-law function

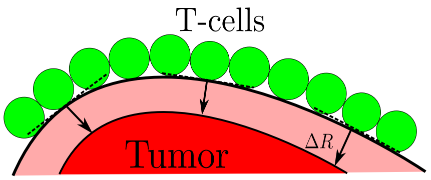

Figure 1. Lymphocytes on the surface of a tumor. Assuming that the tumor (red) has a smooth boundary, the lymphocytes (green) erase a layer of the tumor (light red) in the normal direction to its boundary, at each step.

does not rigorously hold. We can obtain the deviation from this type of decay for a complex morphology. The Equation (5) can be written as

$$\dot{A}(t) = -d(t)A^{1/2}, \tag{11}$$

where

$$d(t) = \sqrt{2}c\frac{\int_0^{2\pi} R(\theta,t)d\theta}{\sqrt{\int_0^{2\pi} R^2(\theta,t)d\theta}} = 2\pi^{1/2}c\sqrt{\frac{L^2(t)}{4\pi A(t)}}. \tag{12}$$

Again, we have assumed that the variations of the radius with respect to the angle are small compared to the size of the radius for all times. Therefore, the function $\delta(t)$ presented in the second section is approximated as

$$\delta(t) = \sqrt{\frac{L^2(t)}{4\pi A(t)}}. \tag{13}$$

A three-dimensional version of the inverse of this parameter has been called the sphericity [3]. For a planar set, the sphericity measures the deviations of a geometrical objet from the spherical shape, in terms of the ratio between the area A enclosed by a curve and the length of its squared perimeter L. It takes a maximum value of one for a disk, and it would take a value of zero for a fractal closed curve, as for example the Koch snowflake, which is made out of three Koch curves [4] placed on the sides of an equilateral triangle. However, we have assumed differentiability.

If the tumor is not convex, then it might occur that during the process of lysis it becomes disconnected. For a disconnected tumor the decay law can be also obtained following the same recipe. But now we have to be more careful, since once a piece has been eliminated, this piece does not contribute any more to the decay. This difficulty can be circumvented by introducing Heaviside step functions. The decay of a disconnected tumor formed by N pieces can be approximated by

$$A(t) = \sum_{i=1}^{N} \left(1 - \Theta(t-\tau_i)\right) A_i(t), \tag{14}$$

where each function $A_i(t)$ is as in Equation (8), τ_i can be computed from Equation (9) and $\Theta(t-\tau_i)$ is the Heaviside step function. The time it takes a disconnected tumor to decay is $\tau = \max \tau_i$.

Finally, it may well happen that the velocity with which the radius decreases is not isotropic, depending on the different values of the angle. Or it may also occur that the velocity at which the radius decreases is not constant in time. In such a case, the best we can do is to provide a partial differential equation relating the variations of the area in time and angle and those of the radius in time

$$\frac{\partial^2 A}{\partial t \partial \theta} = R\frac{\partial R}{\partial t}. \tag{15}$$

Given $R(\theta, t)$, this inhomogeneous second order linear PDE can be integrated to obtain the variations of the area in space and time. For example, we can consider that the velocity with which the radius decreases is anisotropic but constant in time. Then $R(\theta, t) = R_0(\theta) - c(\theta)t$, and again a parabolic decay results, but with a velocity and an acceleration averaged over the different angles.

Acknowledgments

This work was supported by the Spanish Ministry of Economy and Competitiveness under project number FIS2013-40653-P.

References

1. Galilei G. Discorsi e dimostrazioni matematiche, intorno à due nuove scienze attenenti alla mecanica & i movimenti locali. 1638. Leiden: Elsevier, 1904.

2. Osserman R. The isoperimetric inequality. Bull Amer Math Soc. 1978;84: 1182-1238.

3. Wadell H. Volume, shape and roundness of quartz particles. J Geol. 1935;43: 250-280.

4. Koch H von. Sur une courbe continue sans tangente, obtenue par une construction géometrique élémentaire. Arkiv för Matematik Astronomi och Fysik 1. 1904; 681-704.
